# PD-1 Inhibitors in Elderly and Immunocompromised Patients with Advanced or Metastatic Cutaneous Squamous Cell Carcinoma

**DOI:** 10.3390/cancers15164041

**Published:** 2023-08-10

**Authors:** Alexander Yakobson, Ashraf Abu Jama, Omar Abu Saleh, Regina Michlin, Walid Shalata

**Affiliations:** 1 The Legacy Heritage Cancer Center & Dr. Larry Norton Institute, Soroka Medical Center, Ben Gurion University, Beer Sheva 84105, Israelreginam2@clalit.org.il (R.M.); 2 Dermatology and Venereology, The Emek Medical Centre, Afula 18341, Israel

**Keywords:** cemiplimab, pembrolizumab, squamous cell carcinoma, cutaneous, immunotherapy

## Abstract

**Simple Summary:**

This article assesses the efficacy and safety of cemiplimab and pembrolizumab within a complex cohort of cancer patients diagnosed with locally advanced cutaneous squamous cell carcinoma or metastatic cutaneous squamous cell carcinoma. This cohort encompassed individuals with immunosuppressive conditions such as transplant recipients, hematological disorders, and relevant comorbidities, and it included elderly cancer patients. Notably, these specific populations have traditionally been excluded from clinical trials. As a result, our aim is to present the insights garnered from our medical center, highlighting the effectiveness and safety of PD-1 inhibitors in the treatment of advanced cSCC of the skin among elderly and immunosuppressed patients.

**Abstract:**

Cutaneous squamous cell carcinoma (cSCC) of the skin is the second most common form of skin cancer, with aging and prolonged exposure to ultraviolet rays being the main causes of the disease. Cemiplimab and pembrolizumab recently gained regulatory approval for the treatment of locally advanced and metastatic cSCC—conditions that are not treatable by surgical resection and/or radiotherapy. Although the results from the clinical trials have been promising, these studies have not included immunosuppressed, elderly patients. In this study, we included all immunocompromised and immunocompetent patients over the age of 75 years diagnosed with locally advanced or metastatic cSCC and treated with cemiplimab or pembrolizumab. The median duration of follow-up from cSCC diagnosis was 35.6 months, 82.9% of patients were male, and the median age was 83 years old. The median progression-free survival was 8.94 months. The incidence of treatment-related adverse events was 85.6%, the majority of which were grades 1 or 2. The disease control rate was 91.4%, the complete response rate was 17.1%, the partial response rate was 51.4%, the stable disease rate was 23%, and the progressive disease rate was 8.7%. Based on this study, cemiplimab and pembrolizumab for the treatment of locally advanced or metastatic cSCC in elderly, immunocompromised patients are efficacious, with acceptable safety profiles.

## 1. Introduction

Cutaneous squamous cell carcinoma (cSCC) is a type of cancer that affects the squamous cells, which are the flat cells in the outer layer of the skin [1,2,3]. It is the second most common type of skin cancer (15–20%) after basal cell carcinoma (75–80%), with an estimated world-wide incidence of close to 30/100,000 persons [2,3].

The risk of cSCC metastases in the immunocompetent population can range from 0.1% to 9.9%, with a 2.8% chance of mortality due to the disease. The majority of cSCC cases are classified as low-risk. However, for high-risk cSCC, the metastatic rate can reach up to 37%. It is noteworthy that approximately 90% of cSCC metastases manifest within 2 years after the initial diagnosis. Of the patients with cSCC metastases, more than 60% die due to locally invasive cSCC or nodal metastases rather than distant organ metastases. This emphasizes the significance of identifying and addressing the risk factors and potential metastatic spread of the disease at an early stage [4]. The risk factors for recurrence or metastasis of cutaneous squamous cell carcinoma are a Breslow thickness of >2 mm, invasion beyond, subcutaneous fat, perineural invasion, a diameter of >20 mm, poor differentiation, immunosuppression, and a location on the lip, ear, or temple [5].

The prognosis for superficial disease is excellent, with a 5-year survival rate of over 90%. Advanced cSCC represents approximately 5–7% of all cSCC cases [6,7].

The primary causes of cSCC are aging and prolonged exposure to ultraviolet (UV) radiation. People with fair skin, those who sunburn easily, and those who have had significant sun exposure over their lifetime are at higher risk for developing cSCC. Other risk factors include a depleted immune system, a history of skin damage, exposure to certain chemicals, certain viral infections, chronic ulcers, chronic use of steroids, organ transplantation, immunosuppressive treatment, and auto-immune disease [3,8].

The current standard of care for local cSCC is surgical resection, followed by adjuvant radiotherapy in the event of positive surgical margins or in the presence of adverse prognostic factors, such as perineural/perivascular involvement, a large tumor size, a poorly differentiated tumor, or recurrent disease. In addition, definitive radiotherapy may be considered for unresectable lesions or in cases where surgery could result in significant cosmetic or functional morbidity. In some cases, patient preference is an important consideration regarding the decision to forego surgery in favor of radiotherapy [7,9,10].

Approximately 5–7% of cSCC patients are ineligible for local excision or definitive radiotherapy due to unresectable, locally advanced, or metastatic disease [6].

Cemiplimab and pembrolizumab are monoclonal antibodies targeting the PD-1 receptor, and they recently gained regulatory approval for treating locally advanced cutaneous squamous cell carcinoma (lacSCC) and metastatic cutaneous squamous cell carcinoma (mcSCC) patients who are not eligible for curative surgery or radiotherapy. Promising results from phase I and II studies have shown significant antitumor activities by both cemiplimab and pembrolizumab in approximately half of the studied patients, with objective response rates of 35–60% in patients with inoperable or metastatic disease, irrespective of PDL1 expression or genetic mutation burden, and with acceptable safety profiles [11,12]. 

It is important to note, however, that all of these clinical trials excluded patients with immunosuppressive conditions, such as transplant recipients and individuals with hematological diseases, relevant comorbidities, or organ function alterations, all of which are commonly observed in the elderly population. These clinical characteristics which contribute to the frailty of older cancer patients are associated with increased risks of poor therapeutic outcomes.

Therefore, real-world observational studies are important for this population of patients. The aim of this report is to describe the experience of our medical center in the treatment of elderly and immunosuppressed advanced cSCC patients with PD-1 inhibitors.

## 2. Materials and Methods

### 2.1. Patient Selection

This was a single-institution, retrospective observational study. Patients were identified through the electronic medical records at Soroka Medical Center, and we included all immunocompromised patients over the age of 75 years diagnosed with laCSCC or mCSCC and treated with immunotherapy (IO) cemiplimab or pembrolizumab, with initial visits to the Oncology Center between January 2020 and December 2022. The cut-off date for follow-up was April 2023. 

### 2.2. Clinical Data 

The collected patient data included treatment regimen, start and end date of therapy (duration of therapy), date of last follow-up, date of death, date of documentation of disease progression, overall response rate (ORR), and toxicities, and it also included the patients’ Eastern Cooperative Oncology Group (ECOG) performance status (PS) evaluations, which comprised comprehensive medical and therapeutic histories. The evaluations of responses were carried out based on the presence of at least one measurable target lesion. This included observable cutaneous squamous cell carcinoma (CSCC) lesions that were documented during follow-up or via assessable lesions identified by radiological imaging through the assessments of responses which were carried out by the treating oncologist using the immune-related Response Evaluation Criteria in Solid Tumors (iRECIST) criteria. The response outcomes were classified into the following four categories: complete response (CR), partial response (PR), stable disease (SD), and progressive disease (PD). The disease control rate (DCR) was calculated as the percentage of patients who achieved either CR, PR, or SD. The clinical complete response (cCR), partial response (cPR), stable disease (cSD), and disease progression (cPD) were determined based on physical examinations conducted by the attending oncologist. The safety profiles were evaluated by recording the incidence of treatment-related adverse events according to the Common Terminology Criteria for Adverse Events (CTCAE) (version 5.0) [13]. Prior to treatment, all the patients underwent disease staging which involved a total body computed tomography (CT) scan or positron emission tomography–computed tomography (PET-CT). In addition, before receiving their first cycles of treatment, the patients underwent baseline laboratory tests to assess their main organ functions and regulatory parameters. These included complete blood cell counts (including the levels of hemoglobin, leukocytes, neutrophils, lymphocytes, monocytes, platelets, and eosinophils), renal function markers (serum creatinine and blood urea nitrogen), liver function markers (aspartate aminotransferase, alanine aminotransferase, and total bilirubin), albumin, alkaline phosphatase, and thyroid function markers (TSH, T3, and T4), as well as tests for viral infections such as hepatitis B, hepatitis C, and human immunodeficiency virus (HIV).

Throughout each treatment cycle, the same tests were performed as a part of standard laboratory care, and the main purpose of these regular tests was to promptly detect any immune-related adverse events during the course of treatment; however, for the patients not requiring reassessments for hepatitis, HIV, ACTH, and cortisol levels, these specific tests were excluded during subsequent treatment cycles, and radiologic reassessments (CT or PET-CT) were performed every 3 months. 

The study was approved by the Institutional Review Board of Soroka Medical Center (approval no. 0269 on 26 October 2022).

### 2.3. The Inclusion Criteria for the Study 

The inclusion criteria for the study were:

1. Individuals aged 75 years or older who were either immunocompromised or immunocompetent at initial treatment
2. Patients who were diagnosed with unresectable cSCC and who had no response to or were ineligible for radiotherapy (due to the radiation fields or contraindications), patients with locally advanced cSCC, or patients with histologically confirmed metastatic cSCC
3. Patients who were treated with cemiplimab or pembrolizumab as first line therapies and who had no response to or were ineligible for radiotherapy
4. Patients with any Eastern Cooperative Oncology Group (ECOG) performance-status score (0 to 4)
5. Patients with no previous treatment (naïve patients) or who were at least 1 year free of treatment for SCC if they had received previous systemic therapy (to ensure more accurate results that were not influenced by prior treatments)
6. Patients who had complete follow-up histories in the Soroka Medical Center records.

Each study patient, on admission to the Oncology Center at Soroka Medical Center, was presented to a multidisciplinary medical team that included a medical oncologist, radiation oncologist, dermatologist, plastic surgeon, pathologist, and radiologist. Each patient was assigned a primary physician who was responsible for managing the patient during the course of treatment.

At our center, patients with locally advanced or metastatic diagnoses are treated mainly by medical oncologists, and the treatment plans are generally based on National Comprehensive Cancer Network (NCCN) recommendations [14].

**Figure 1:**
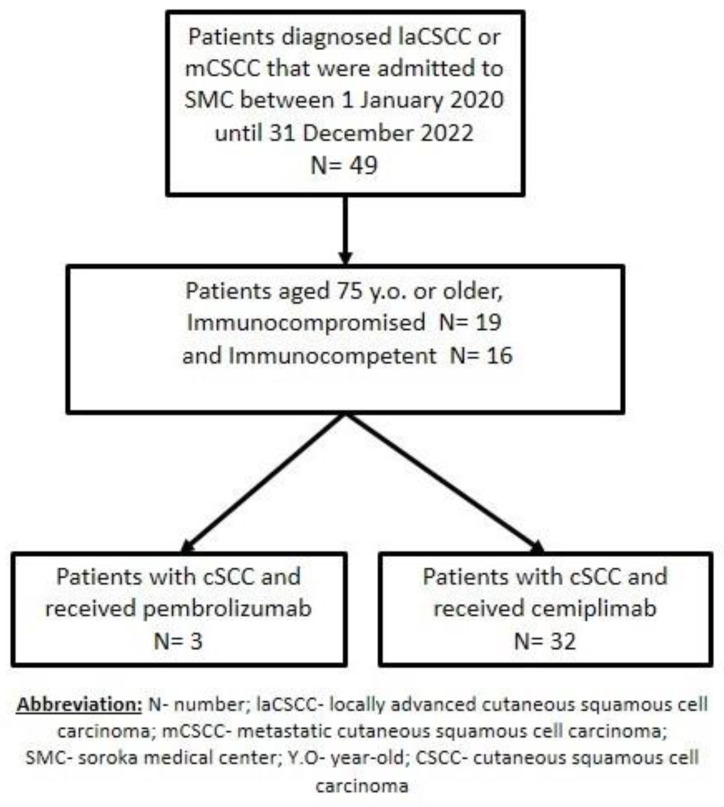
The workflow used for our retrospective observational study of patients with locally advanced cutaneous squamous cell carcinoma or metastatic cutaneous squamous cell carcinoma who were treated with immunotherapy.

Forty-nine patients were screened, and forty-nine patients met the eligibility criteria for this study and were included in the analysis (fourteen were excluded because they did not receive IO or were younger than 75 years) (Figure 1).

### 2.4. Treatment Administered

Cemiplimab was administered at a uniform dose of 350 mg intravenously every 21 days until disease progression commenced or unacceptable toxicity levels were reached [11].

Pembrolizumab was administered at a uniform dose of 200 mg intravenously every 21 days until disease progression commenced or unacceptable toxicity levels were reached [12].

## 3. Results

Of the 35 eligible patients for this study, all were evaluated for their responses and safety. The patient characteristics at baseline are summarized in Table 1. The median duration of follow-up from the time of cSCC diagnosis was 35.6 months. Most of the patients were male (82.9%). The median age of the patients in our study was 83 years (range of 75–98), with the median age for the females being 91 years (range of 76–98) while the males’ median age was 81 years (range of 75–96).

**Figure 2:**
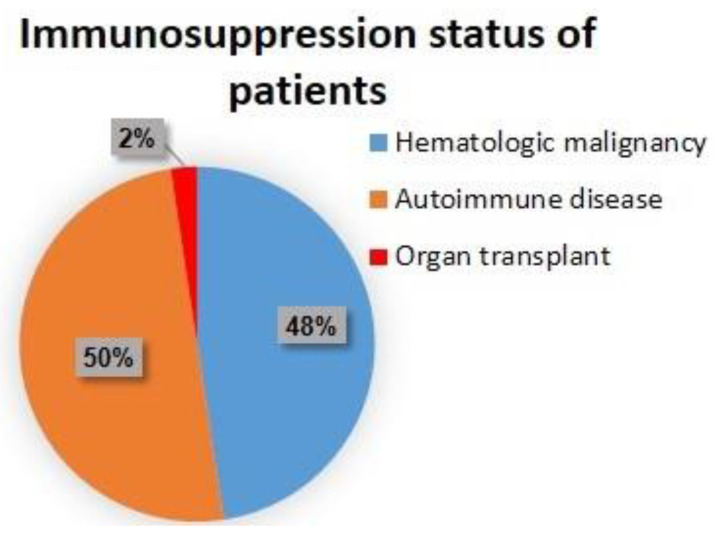
Immunosuppression status of the patients at baseline.

There were slightly more immunocompetent than immunocompromised patients (19 (54.29%) vs. 16 (45.71%)) (Figure 2).

The group for immunosuppression consisted of organ transplants (kidney). The hematologic malignancies included two patients with polycythemia vera and two patients with chronic lymphocytic leukemia, marginal zone lymphoma, gastric MALT lymphoma, and multiple myeloma. The patients with autoimmune diseases comprised two patients with psoriasis and two patients with rheumatoid arthritis, inflammatory bowel disease, polymyalgia rheumatica, and immune thrombocytopenia.

The overall ECOG performance status was fairly good, with no patients having an ECOG score of three or four. In our cohort, the most common stage was three, comprising almost half of the patients (48.6%). In our cohort, 62.9% of the patients had previously received treatment (surgery (22.9%), radiotherapy (17.2%), surgery followed by radiotherapy (14.3%), immunotherapy (5.7%), and chemotherapy (2.9%)) and 37.1% were treatment naïve. The most common primary tumor site was the scalp, occurring in 34.3% of patients, followed by the face, nose, orbital, or cheeks in 28.6% of the patients. The treatments consisted of cemiplimab in 32 patients and pembrolizumab in 3.

The median progression-free survival (PFS), defined as the time from the start of therapy to the documentation of disease progression or death, in months (range), was 8.94 (2–26). Among the immunosuppressed patients, the PFS was 10.62 months (2–26), which was longer than the median PFS of the immunocompetent patients (7.52 months (2–28)). 

The incidence of treatment-related adverse events (trAEs) of all grades for all patients was 85.6% (30 patients), (Table 2), with no treatment-related deaths. Most of the trAEs reported were grades 1 or 2 (11 patients (31.4%) and 16 patients (54.2%), respectively). During the study and treatment period (up to 42 months of follow-up), nine patients died (four due to disease progression (PD), one due to pulmonary embolism, one due to trauma, one due to myocardial infarction, one due to sepsis, and one due to general deterioration). 

Of the thirty patients that reported trAEs, six patients received steroid therapy for their trAEs and one patient received vedolizumab due to grade four diarrhea. No patients stopped treatment due to trAEs. Furthermore, all patients resumed the treatment of IO after resolution of the trAEs.

**Figure 3:**
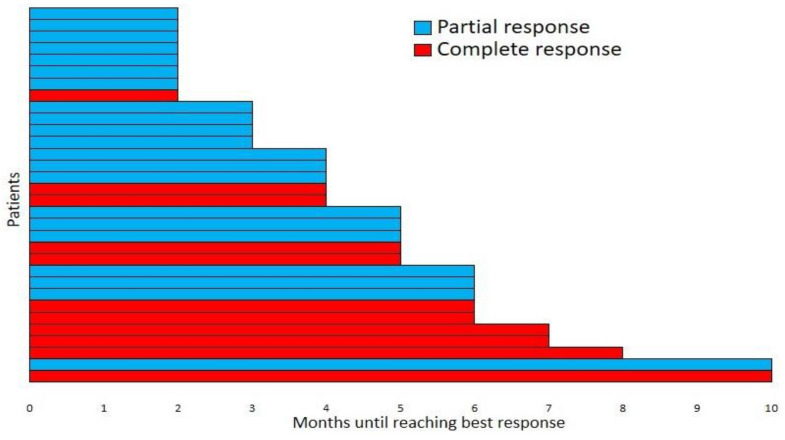
Time to the best responses for the patients not progressing during treatment.

The median time for the best response under IO therapy was 4.5 months (range of 2–10 months) (Figure 3). Among the 35 patients in our study, the disease control rate (DCR (the complete response (CR) rate plus the partial response (PR) and stable disease (SD) rates) was 91.4%. The best responses were the CRs for six patients (17%), including the single patient treated with pembrolizumab; the PRs for eighteen patients (51.3%), including the two patients treated with pembrolizumab; the SDs for eight patients (23%); and the progressive disease (PD) rates for three patients (8.7%) (Table 3).

## 4. Discussion

CSCC remains the second most common type of non-melanoma skin cancer, following basal cell carcinoma [1]. CSCC in its advanced stage, when it cannot be treated with surgery or radiotherapy, is a serious condition that typically affects older and more fragile patients, such as those in our study (patients older than 75 years and immunosuppressed patients). Advanced CSCC can cause significant physical limitations, as well as pain and disfigurement. Managing this condition requires a team approach that encompasses multiple disciplines to achieve positive outcomes and maintain a patient’s quality of life.

The present study describes a real-world single-institution, and it is a retrospective observational study of the use of PD-1 inhibitors for the treatment of cSCC. The results revealed a high DCR with acceptable toxicity levels. All of our patients were treated in an academic facility by experienced medical oncologists and radiotherapists, along with other relevant specialists as part of the disease management team.

While surgery is the standard treatment for resectable cSCC, recent clinical trials have revealed that PD-1 inhibitors could be a valuable addition to such treatment [15]. Cemiplimab has demonstrated the value of a paradigm shift in the treatment of laCSCC and mCSCC. In 2018, Migden et al. conducted a phase I trial of cemiplimab, an immune-checkpoint inhibitor, in an open-label, non-randomized study that included 26 patients with locally advanced or metastatic CSCC. The patients experienced rapid reductions in their tumor volumes within weeks of beginning therapy, with an overall objective response rate (ORR) of 50%. Preceding FDA approval, the results of a phase II trial (the phase 2 EMPOWER trial), including 59 patients with metastatic disease, showed an ORR of 47%, where the duration of response exceeded 6 months in 57% of the responders [13]. Two years later, in 2020, Rischin et al. conducted a non-randomized control trial of 56 patients on a fixed dosing of cemiplimab 350 mg every 3 weeks intravenously, and they documented an ORR of 41%, where the duration of response at 8 months was estimated to be 95% (95% CI 67–99%) [16]. A year later, using pembrolizumab, Hughes et al. observed an ORR of 50% in a phase II trial of 159 patients, where there was an estimated response duration of 12 months or more in 80% of patients [17]. 

High-affinity PD-1 inhibitors such as cemiplimab and pembrolizumab are emerging as promising first-line treatment options for patients with advanced cSCC who are not candidates for curative-intent surgery or chemo-/radiotherapy. Phase II cohort studies have reported objective response rates ranging from 35% to 60% in patients with non-operable or metastatic disease [11,13,16,17,18].

No patients with a significant immunosuppressive condition were included in those studies [12,13,15,16,17,18,19], and therefore, there is limited information on the management of such complex situations that we encounter in real-life practice.

Overall, 85.7% (30 patients) of the patients in our cohort experienced different grades of toxicity, as previously mentioned, with three (8.6%) having grade 3 or higher toxicity levels, but there were no patient deaths due to trAEs. Crucially, we did not identify any discernible differences in the occurrences of irAEs within these groups. Clinical trials such as the KEYNOTE-629, CARSKIN, and the EMPOWER trials have reported varying rates of grade 3–5 adverse events related to treatment-induced toxicity. The KEYNOTE-629 study reported an 11.9% rate of such events, with two deaths (1.3%). Similarly, the CARSKIN trial reported four patients (7%) with grade 3 or higher toxicity, including one related death (2.6%). In the cemiplimab trial, Migden et al. documented treatment-related adverse events in 34 patients (44%), with grade 3–4 toxicity, among whom 1 (1.3%) died [13,16,17,19,20,21]. Regarding our study, there were fewer than expected grade 3 or higher trAEs. Our hypothesis for this finding is that the treatments were conducted by a team experienced in previous IO protocols, with close monitoring. In addition, since most of the patients in our study were in a fragile condition, detecting and treating adverse effects promptly likely resulted in better outcomes. Interestingly, the immunosuppressed elderly patients (those over 75 years old) had a better PFS outcomes (10.6 months) than the immunocompetent patients (7.5 months).

The response for this regimen was better than expected, with a DCR of 91.4%, which was a higher rate than has been seen in previous clinical trials (the DCR for pembrolizumab was 52.4%, and for cemiplimab, it was 61%) [11,12]. Furthermore, two retrospective studies have suggested that elderly patients may experience better benefits compared to their younger counterparts. This finding may be related to the observation of a more favorable antitumor balance of CD8 T cells to regulatory T cells within the tumor microenvironment [22,23,24]. In addition, other investigators have reported responses to PD-1 blockades in patients with cSCC who have undergone kidney transplantation or have leukemia, as well as in patients undergoing immunosuppressive therapy for autoimmune diseases. It has also been demonstrated that immunosuppressed patients have a high likelihood of responding to PD-1 blockades for other cancers [24,25,26,27,28,29].

In solid tumors, the inflammatory response can be characterized by various parameters in the peripheral blood, including baseline leukocytes and their subtypes, C-reactive protein, plasma fibrinogen, the neutrophil-to-lymphocyte ratio, albumin, alkaline phosphatase, and the lymphocyte-to-monocyte ratio. These parameters have been widely discussed as prognostic indicators in many solid tumors, especially in skin cancers (cutaneous melanoma) [30,31,32,33,34,35].

Our retrospective pre-treatment peripheral blood analysis revealed, contrary to what has been reported in other skin cancers, that the values of hemoglobin, albumin, leukocytes, alkaline phosphatase, neutrophils, and eosinophils and the neutrophil-to-eosinophil ratio showed no significant differences in the PFS results, regardless of whether the levels were high, low, or in normal ranges, and these parameters did not influence the incidence of trAEs.

The importance of real-world data in cSCC cannot be overstated given the varied and frequently frail baseline characteristics of this patient population. Patients with cSCC are typically older and have a wide range of comorbidities that may render them ineligible for clinical trials due to factors such as poor performance status (ECOG 3-4), solid organ transplantation, immunosuppressive therapy, hematologic malignancies, and autoimmune diseases. Therefore, a significant proportion of the treatment population lacks data upon which to base treatment decisions. Our study demonstrated that the effectiveness of anti-PD-1 treatment, at least in our small cohort, was not impacted by various forms of immunosuppression, which supports previous research [36,37,38].

PD-1 is expressed on various cell types, including CD4 and CD8 T cells, as well as NK cells, B cells, and macrophages. Typically, its expression is elevated in activated T cells. Upon binding with its ligands—PD-L1/L2 found on hematopoietic and cancer cells—PD-1 facilitates the suppression of T cell responses. This involves the recruitment of SHP-1/SHP-2 phosphatases, resulting in the reduced intracellular signaling of TCR and CD28 alongside the downregulation of downstream transcription factors and T-cell-secreted cytokines. The inhibitory effect of PD-L1/L2 on effector T cell response can be counteracted by competing ligands such as B7 (CD80) and RGMB for PD-L1 and PD-L2, respectively. Furthermore, recent research has indicated that blocking PD-1 on regulatory T cells can lead to the increased activation and expansion of immune-suppressive Treg within the tumor microenvironment. This highlights the potential significance of the balance between PD-1+ Treg and PD-1+ effector T cells within tumors in determining the outcomes of PD-1-directed checkpoint antibody therapy. Moreover, PD-1 blockades may also enhance NK cell activity within tumors and modulate the behavior of innate lymphoid cells (ILC). Some studies have proposed that NK cells play a role in mediating the effects of anti-PD-1 blockades in conditions such as metastatic melanoma and non-small-cell lung carcinoma [39,40,41,42].

While our study provides valuable insights into the safety and efficacy of immunotherapy in elderly patients (those over 75 year old), including elderly immunosuppressed patients with cSCC, we acknowledge that there were several limitations. There was potential bias due to the retrospective data sourced from a single institution and the relatively small sample size. Perhaps because of the small size of the study, we were unable to identify a laboratory predictive factor as found in melanoma, as mentioned before, and we did not identify a correlation between the retrospective blood tests and the outcomes for cSCC. In addition, while exploring alternative treatments and information for cSCC, it is important to acknowledge the limitation posed by the absence of using PDL-1 Inhibitors or tyrosine kinase inhibitors, which are currently not used in the treatment of cSCC. This aspect warrants further verification and investigation. In the future, as other treatments come into play, a comparative analysis of their efficacy and safety levels could provide valuable insights. However, to corroborate these findings, forthcoming investigations should span diverse institutions or nations and involve more extensive groups of patients. Moreover, a requisite expansion of the follow-up duration, mirroring the approach undertaken in the initial study, is imperative for a comprehensive scrutiny of the alterations.

Despite the limitations of our study, it is important as it is, to the best of our knowledge, the first to characterize the safety and efficacy of immunotherapy in elderly and immunosuppressed patients with cSCC. Our findings could have important implications for the management of this patient population. Our study underscores the importance of carefully considering and monitoring immunotherapy in this complex patient group.

## 5. Conclusions

In this real-world experience, despite its limitations, including its observational nature, small patient sample size, and exclusive reliance on data extracted from a single institution in Israel, which prevented the inclusion of data from other ethnicities, we provide valuable evidence supporting the high antitumor activities and safety profiles of cemiplimab and pembrolizumab in a diverse group of patients that were ineligible for clinical trials. Additionally, our study sheds light on the efficacy of cemiplimab and pembrolizumab in fragile and octogenarian immunocompromised patients.

## Figures and Tables

**Table 1:** Demographic and disease characteristics of the patients at baseline.

**Age** years, median (range)	83 (75–98)
Male	81 (75–96)
Female	91 (76–98)
**Sex**	
Male	29 (82.85%)
Female	6 (17.15%)
**Immunosuppression status, N (%)**	
Immunocompetent	19 (54.29%)
Immunosuppressed	16 (45.71%)
Autoimmune disease	8 (22.85%)
Hematologic malignancy	7 (20%)
Organ transplant	1 (2.85%)
**ECOG status**	
0	6 (17.15%)
1	17 (48.57%)
2	12 (34.28%)
**Stage of disease**	
2	8 (22.85%)
3	17 (48.58%)
4	10 (28.57%)
**Treatment Status**	
Treatment naïve	13 (37.14%)
Previous surgery	8 (22.85%)
Previous radiotherapy	6 (17.15%)
Previous surgery followed by radiotherapy	5 (14.28%)
Previous immunotherapy	2 (5.71%)
Previous chemotherapy	1 (2.85%)
**Location of primary tumor**	
Scalp	12 (34.28%)
Face (face, nose, orbital, or cheeks)	10 (28.57%)
Ear	3 (8.57%)
Upper limbs, trunk, or back	3 (8.57%)
Lower limbs	3 (8.57%)
Unknown primary	2 (5.71%)
Penile	1 (2.85%)
Neck	1 (2.85%)

**Table 2:** Adverse event profiles.

Type of Toxicity	Number of Patients (%)	Grade 1	Grade 2	Grade 3	Grade 4
Fatigue	13 (37.1%)	3	9	1	-
Rash	7 (20%)	4	3	-	-
Thrombocytopenia	3 (8.6%)	-	3	-	-
Myalgia	3 (8.6%)	3	-	-	-
Hypothyroidism	1 (2.9%)	1	-	-	-
Cerebral arthritis	1 (2.9%)	-	-	1	-
Constipation	1 (2.9%)	-	1	-	-
Diarrhea	1 (2.9%)	-	-	-	1

**Table 3:** Disease control rates (N (%)).

Complete response	6 (17%)
Partial response	18 (51.3%)
Stable disease	8 (23%)
Progressive disease	3 (8.7%)

## Data Availability

The data are contained within the article or are available from the authors upon reasonable request.
